# PRX1-positive mesenchymal stem cells drive molar morphogenesis

**DOI:** 10.1038/s41368-024-00277-0

**Published:** 2024-02-19

**Authors:** Xiaoqiao Xu, Xuyan Gong, Lei Zhang, Han Zhang, Yao Sun

**Affiliations:** Department of Implantology, Stomatological Hospital and Dental School of Tongji University, Shanghai Engineering Research Center of Tooth Restoration and Regeneration, Shanghai, China

**Keywords:** Mesenchymal stem cells, Morphogen signalling

## Abstract

Mammalian teeth, developing inseparable from epithelial-mesenchymal interaction, come in many shapes and the key factors governing tooth morphology deserve to be answered. By merging single-cell RNA sequencing analysis with lineage tracing models, we have unearthed a captivating correlation between the contrasting morphology of mouse molars and the specific presence of PRX1^+^ cells within M1. These PRX1^+^ cells assume a profound responsibility in shaping tooth morphology through a remarkable divergence in dental mesenchymal cell proliferation. Deeper into the mechanisms, we have discovered that *Wnt5a*, bestowed by mesenchymal PRX1^+^ cells, stimulates mesenchymal cell proliferation while orchestrating molar morphogenesis through WNT signaling pathway. The loss of *Wnt5a* exhibits a defect phenotype similar to that of siPrx1. Exogenous addition of WNT5A can successfully reverse the inhibited cell proliferation and consequent deviant appearance exhibited in *Prx1*-deficient tooth germs. These findings bestow compelling evidence of PRX1-positive mesenchymal cells to be potential target in regulating tooth morphology.

## Introduction

Mammalian tooth, which is arising from a tooth germ, is an important organ involved in food chewing and digestion, pronunciation and maintaining facial fullness.^1,2^ The past decades of research have greatly improved our understanding of tooth development, regeneration, repair and evolution.^3,4^ However, the key factors and cell types regulating tooth morphology remain to be revealed. Differ from other ectodermal organs, teeth come in many shapes.^5^ For example, there are four main shapes in human teeth: incisors, canines, premolars and molars, while only incisors and molars are existent in mouse upper and lower jaws. The tooth germs undergo a strict regulatory process for different morphogenesis, which ultimately ensures that the tooth forms the correct shape in the correct position.^6^ Thus, mammalian tooth, which is arising from a tooth germ, has also become an important model responsible for the studies of organ morphogenesis. Notable among them were the molars, the morphology of which varied according to different positions.^7^ Remarkably, mouse first molar (M1) and human molars are similar in shape, whereas the third molar (M3) has a completely different shape from M1 and resembles a human premolar. Why do the molars have different shapes? Its regulation mechanism deserves further work for elaboration.

At around embryonic day 11.5 (E11.5), molar development initiates by local thickening of the dental epithelium at the putative tooth site and proliferation to form a tooth bud. Accompanied by underlying mesenchymal condensation, original odontogenic mesenchymal stem cells are derived, which in turn form the tooth germ through epithelial-mesenchymal interaction.^4^ Mesenchymal stem cells, known as MSCs, are stromal stem cells capable of self-replication, the ability to differentiate into multiple lineages, and the power of regeneration,^8^ and are closely related to tooth development as well. More importantly, the odontogenic potential, which is capable to induce gene expression of adjacent tissue to initiate tooth development and morphogenesis, has already switched to the mesenchyme at the early bud stage (E12.5), and resides in the dental papilla till birth.^9^ Tissue engineering experiments have also proved that the shape of a heterotopic reassociation tooth depends on the origin of the mesenchyme.^10^ While these dental MSC populations share common characteristics, they also exhibit a fascinating heterogeneity. One compelling piece of evidence lies in the diverse spatial localizations of MSCs orchestrate the construction of dentin, cementum, dental pulp, and the periodontal ligament (PDL).^11^ In recent years, the utilization of genetic lineage-tracing techniques, coupled with the exploration of single-cell transcriptomics, has made great progress to identify a range of mesenchymal cell subpopulations in vivo,^12^ suggesting possible divergence with a focus on development. However, whether tooth morphology is determined by these heterogeneous MSC subpopulations, the precise roles they play in the intricate process of tooth morphogenesis beckon further investigation. Therefore, unraveling the key subsets of MSCs involved in the regulation of tooth morphology represents one of the central scientific challenges in this field.

PRX1 (also known as PRRX1), paired-related homeobox-1, stands as an iconic marker of mesenchymal stem cells.^13^ Extensive research has demonstrated the pivotal role played by PRX1-positive cells in diverse developmental processes, including bone formation and tooth development, among others.^14,15^ Notably, during the orchestration of tooth morphogenesis, PRX1 transcripts are widely expressed within the undifferentiated mesenchyme that precedes molar formation. These PRX1-positive cells are considered as important stem cells in the progression of tooth development. At the cell differentiation stage, the PRX1-positive cell populations differentiate into specific odontogenic cells that actively contribute to the formation of dentin, pulp, periodontal ligament, and alveolar bone.^16^ Astonishingly, our previous investigations have given out that the lineage of PRX1-positive cells serves as the source of the mesenchyme for the mouse first molar, while the origin of mesenchymal cells for the mouse third molar with smaller size and fewer cusps remains independent of the PRX1 lineage.^17^ Consequently, this finding signifies that the heterogeneity exhibited by dental MSCs can exert certain influences on the process of tooth morphogenesis, with PRX1-positive cells occupying a critical position worthy of in-depth exploration.

In this study, we identified heterogeneity within the dental mesenchymal stem cell (MSC) subpopulations, PRX1 subgroup. We sought to elucidate the functional roles of PRX1-positive MSCs in tooth morphology regulation, and found the specific expression of the morphogen *Wnt5a*, a key regulator governing the proliferation and bidirectional crosstalk of epithelial-mesenchymal interactions. We observed a link between PRX1-positive cells and M1 mesenchymal cellular proliferation, fostered by the influence of *Wnt5a*. Remarkably, the absence or depletion of PRX1-positive cells or *Wnt5a* manifested as alterations in molar morphology, and exogenous addition of *Wnt5a* agonist could partially reverse the inhibited cell proliferation and consequent dental morphological changes caused by PRX1 deficiency. Collectively, our results underscore the pivotal role played by PRX1-mediated mesenchymal stem cell subpopulations in driving the diversity in the process of tooth morphogenesis with therapeutic potential and far-reaching implications for regenerative dentistry.

## Results

### Dental PRX1 expression correlated with tooth morphogenesis

**Fig. 1:**
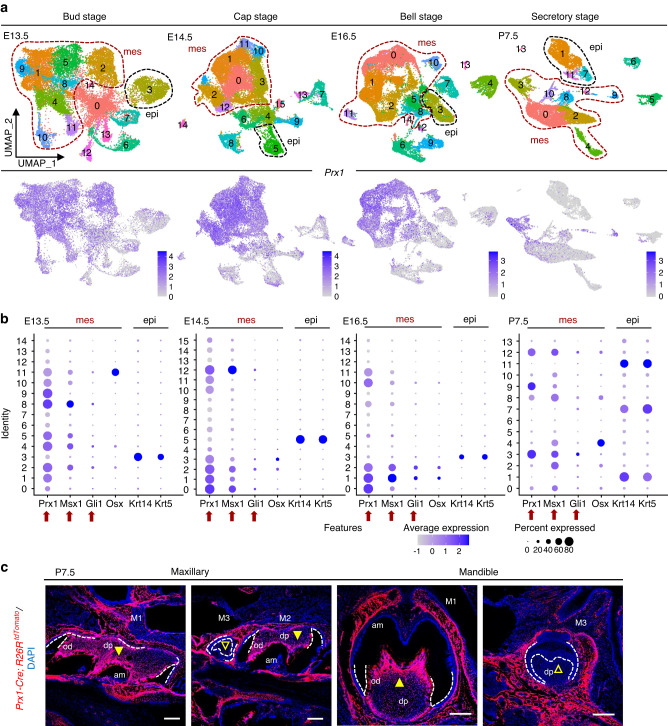
Distribution and expression of PRX1 in molar tooth germs. **a** *Prx1* expression in integrated molar germs from four stages via UMAP. Epithelial and mesenchymal lineages were separated by classic markers respectively, including *Msx1, Gli1* for mesenchyme and *Krt14, Krt5* for Epithelium. epi epithelium, mes mesenchyme. **b** Correlation between PRX1-positive cells and dental mesenchyme or epithelium at from bud stage to secretory stage. The PRX1-positive cells distributed mostly in the condensed mesenchyme of developing molar, especially at the early morphogenetic bud through the cap stages. epi epithelium, mes mesenchyme. **c** Distribution of PRX1-positive cells in M1-M3 of the maxillary and mandible at P7.5 (*n* = 3). The triangles indicate areas of mesenchymal origin. The dotted line marks the edge of tooth germ and the boundary between epithelium and mesenchyme. PRX1^+^ cells and their progeny (red) were confined to the pulp of M1 and M2, but were rarely distributed in M3. M1 the first molar, M2 the second molar, M3 the third molar, od odontoblast, am ameloblast, dp dental pulp. Scale bar: 100 μm

A few previous studies have demonstrated the plasticity of dental stem cells in differentiation patterns, which previously thought to be homogeneous stem cell populations are actually highly heterogeneous.^18,19^ Among them, PRX1 is a mesenchymal stem or progenitor cell marker commonly used in craniomaxillofacial development research.^15^ To determine a function for *Prx1*, we re-analyzed the published single-cell transcriptomes during mouse first molar development from E13.5 to P7.5. Compared with other stem/progenitor cell markers commonly used during tooth development studies, *Prx1* was specifically expressed in the condensed mesenchyme of developing molar, especially at the early morphogenetic bud through the cap stages, but is down-regulated once differentiation occurs at the bell stage (Fig. 1a, b). This preliminarily indicates the correlation between PRX1 and tooth morphogenesis. Meanwhile, lineage tracing technique is an important tool to study the characteristics of stem cells during mammalian tissue development.^20^ The result of tracing partially revealed the similarity and heterogeneity of dental MSCs. To further investigate the role of PRX1 in molar development, PRX1^+^ cells were first traced during tooth development. It turned out that PRX1^+^ cells and their progeny were confined to the condensed mesenchyme of the first and second molars during development, but were rarely distributed during the morphological development of M3 tooth germ (Fig. 1c, Fig. S1). More interestingly, in line with the apparent morphological differences between the first and third molars during natural tooth development in mice, we speculated that PRX1-positive MSCs might be closely related to tooth morphological determination.

### PRX1 deficiency in third molar induces morphological differences and retarded proliferation of dental MSCs

**Fig. 2:**
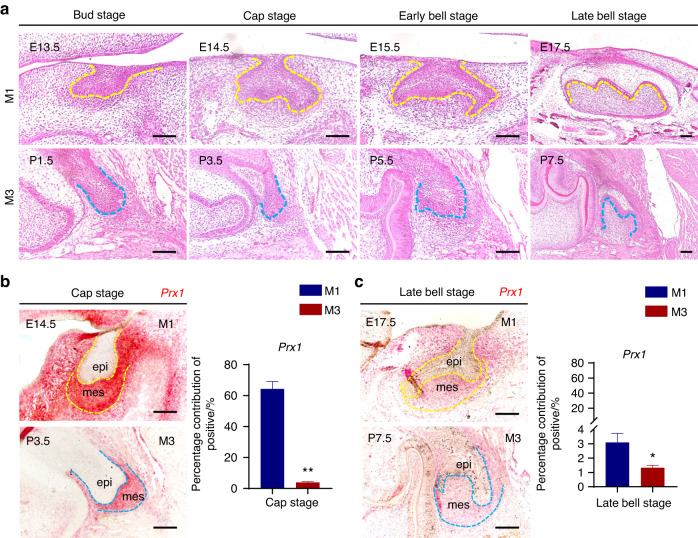
Differential PRX1 expression caused morphological diversity between M1 and M3. **a** H&E staining of tooth germs in the sagittal plane showing the morphological differences between M1 and M3 during development. The dotted line marks the boundary between epithelium and mesenchyme. Scale bar: 100 μm. **b** Representative images and quantification of in-situ *Prx1* expression at cap stage of M1 and M3. The dotted line marks the boundary between epithelium and mesenchyme. epi epithelium, mes mesenchyme. Scale bar: 100 μm. The error bar represents the standard deviation of the mean. **P < 0.01. **c** Representative images and quantification of in-situ *Prx1* expression at late bell stage of M1 and M3. The dotted line marks the boundary between epithelium and mesenchyme. epi epithelium, mes mesenchyme. Scale bar: 100 μm. *P < 0.05

Above results suggested that the differential distribution of PRX1 may be the cause of the different morphology of M1 and M3. Therefore, we then focused on the morphological differences between M1 and M3 and their causes. The development of M3 begins perinatally, and is basically complete at about 4 weeks. From the coronal view, the development pattern of M3 tooth germ is similar to that of M1 tooth germ, which also goes through bud, cap and bell stages of morphogenesis (Fig. S2a). The difference in M3 is that, the epithelial cap and dental papilla were not very pronounced as in M1, so much so that the epithelium was not as stretched out as M1 and considerably smaller during the bell stage. The greater morphological differences are mainly reflected in the sagittal plane. During the transition from cap stage to bell stage of M3 tooth germ, there is visible less increase in the number of mesenchymal cells and a noticeable reduction in the number of cuspal regions compared to M1 (Fig. 2a). The dynamic changes in *Prx1* mRNA expression during M1 development were verified by RT-qPCR (Fig. S2b), which was consistent with the results of single-cell data analysis. Meanwhile, *Prx1* mRNA expression levels between M1 and M3 verified by RT-qPCR indicated the specific presence of PRX1^+^ cells within M1 (Fig. S2c). Also, RNAscope was performed to further verify the different *Prx1* expression between M1 and M3. The results showed that *Prx1* RNA was also expressed in M3, but both quantity and intensity were significantly lower than *Prx1* in M1 (Fig. 2b, c), which was also consistent with the results detected by RT-qPCR.

**Fig. 3:**
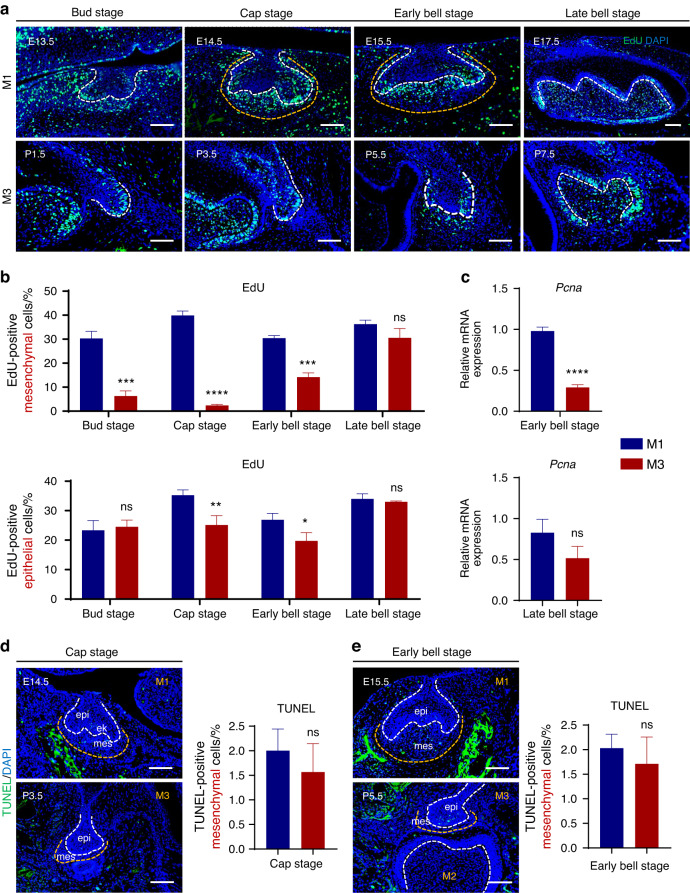
PRX1 deficiency in vivo induced retarded proliferation in M3. **a** Representative images of EdU labelled proliferating cells (green) in tooth germs at different developmental stages. Samples were collected 2 h after EdU administration (*n* = 3 per developmental stages). Scale bar: 100 μm. **b** Quantification of EdU-positive cells in dental mesenchyme and epithelium between M1 and M3 at bud, cap and bell stages (*n* = 3 per developmental stages). The error bar represents the standard deviation of the mean. ns no statistical significance, *P < 0.05, **P < 0.01, ***P < 0.001, ****P < 0.000 1. **c** *Pcna* mRNA expression between M1 and M3 was verified by RT-qPCR. M3 showed significantly lower *Pcna* expression in early bell stage. ns no statistical significance, ****P < 0.000 1. **d** Representative images and quantification of TUNEL labelled apoptotic cells (green) in tooth germs of M1 and M3 at cap stage (*n* = 3 per developmental stages). Scale bar: 100 μm. ns no statistical significance. **e** Representative images and quantification of TUNEL labelled apoptotic cells (green) in tooth germs of M1 and M3 at early bell stage (*n* = 3 per developmental stages). Scale bar: 100 μm. ns no statistical significance

To further explore the reasons for the difference in molar morphology between M1 and M3, we first tested cell proliferation of both M1 and M3 at the same developmental stages by Edu labeling. It was found that the proliferation of mesenchymal cells in developing M1 was much more active than in M3, while the proliferation of epithelial cells in M1 was slightly more than in M3 at cap and early bell stage (Fig. 3a, b). The mRNA level of cell proliferation marker *Pcna* also showed that cells of M3 were at a lower proliferation level compared to M1 (Fig. 3c). Besides, TUNEL assay was conducted to assess the rate of apoptotic cells of tooth germs from M1 and M3. Most of the apoptotic cells between the two were found at the site of enamel knots in the epithelium, while there were fewer apoptotic cells in the mesenchyme of M1 and M3, and there was no significant difference between them (Fig. 3d, e). The above results suggest that differences in the proliferation of mesenchymal cells, which was mediated by PRX1, may influence the number of cusps. Consequently, in line with the apparent morphological differences between the first and third molars during natural tooth development in mice, we further determined that PRX1-positive MSCs were closely contribute to tooth morphological determination.

Besides, we checked the odontogenic differentiation capacity of M1 and M3 mesenchymal cells. Osterix (Osx/Sp7) is a specific transcription factor committed to the dentin-forming odontoblastic lineage.^21^ The expression levels of Tomato^+^ cells from *Osx-cre; R26R*^*tdTomato*^ mice verified that OSX was highly expressed during odontoblast differentiation of dental MSCs, both M1 and M3 (Fig. S3a). Immunofluorescence staining showed that the typical markers of odontogenic differentiation, OSX and RUNX2, were expressed in both molars (Fig. S3a, b). Therefore, it indicated that odontogenic differentiation capacity of MSCs in both M1 and M3 was not determined by PRX1^+^ cells.

### PRX1 knockdown in vitro inhibited cell proliferation and mesenchymal pluripotency

**Fig. 4:**
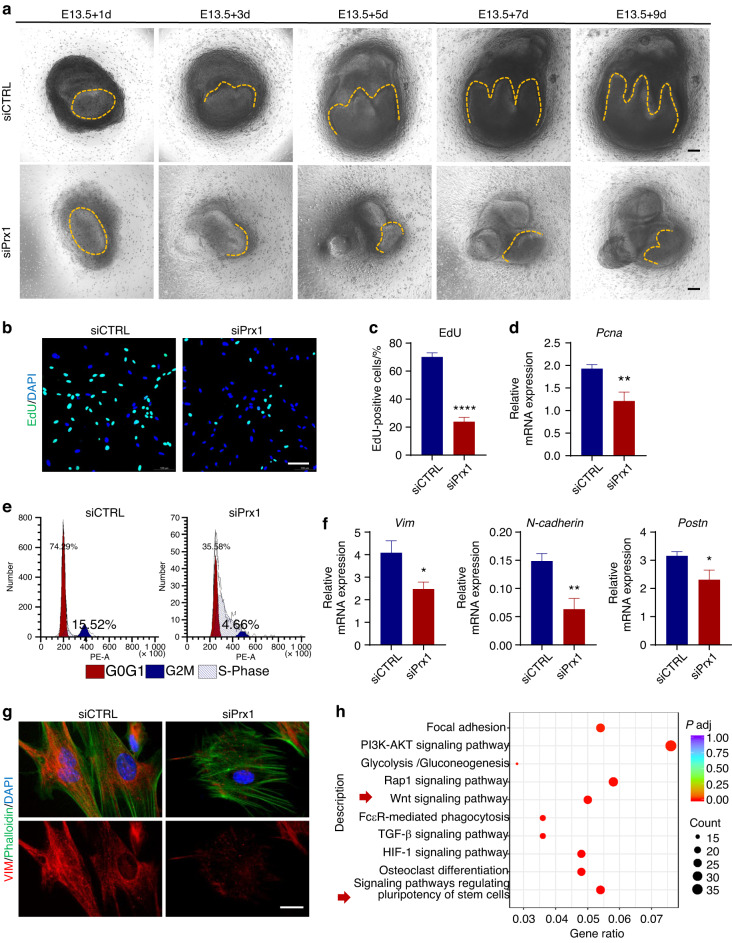
PRX1 knockdown in vitro altered M1 morphology, cell proliferation and mesenchymal pluripotency. **a** In vitro culture images of M1 germs at 1, 3, 5, 7 and 9 days after transfection of *Prx1* siRNA (siPrx1) or negative control (siCTRL). Scale bar: 100 μm. **b** Confocal images of EdU labelled proliferating dental MSCS after transfection of *Prx1* siRNA (siPrx1) or negative control (siCTRL). Samples were collected 12 h after EdU administration (*n* = 3). Scale bar: 100 μm (**c**) Quantification of EdU-positive cells between siPrx1 and siCTRL group (*n* = 3). The error bar represents the standard deviation of the mean. ****P < 0.000 1. **d** *Pcna* mRNA expression between siPrx1 and siCTRL group was verified by RT-qPCR (*n* = 3). it showed significantly lower *Pcna* expression after *Prx1* knockdown. **P < 0.01. **e** Flow cytometry analysis of cell cycle phases to identify the impact of *Prx1* knockdown on cell cycle arrest. **f** mRNA expression level of mesenchymal markers *Vim*, *N-cadherin*, and *Postn* after *Prx1* knockdown (*n* = 3). *P < 0.05, **P < 0.01. **g** Immunofluorescence for VIM (red) and cytoskeleton (Phalloidin, green) was performed to compare the mesenchymal signature and cellular shape after *Prx1* knockdown to negative control. Scale bar: 25 μm. **h** Kyoto encyclopedia of genes and genomes (KEGG) pathway enrichment analysis showed the top ten differentially expressed pathways in M1 tooth germs between siPrx1 and siCTRL group, including signaling pathways regulating pluripotency of stem cells and Wnt signaling

To further confirm the specific role of *Prx1* in tooth germ morphogenesis, both first molar tooth germs and primary cells from dental mesenchyme were cultured in vitro^22,23^ and then *Prx1* expression was knocked down with siRNA. *Prx1* knockdown in vitro resulted in a reduction in the number of molar cusps and smaller teeth (Fig. 4a), indicating that PRX1 was involved in the regulation of tooth germ morphogenesis. Next to find out the biological function of *Prx1* during tooth germ morphogenesis, we also examined whether there was a corresponding change in the level of proliferation of dental mesenchymal cells after *Prx1* knockdown. EdU labeling and *Pcna* mRNA expression showed a significant decrease compared to the negative control group (Fig. 4b–d). Also, cell cycle analysis was performed after *Prx1* knockdown by siRNA. It was found that the number of cells in the G0/G1 phase roughly decreased in the siPrx1 group (Fig. 4e), suggesting that PRX1 knockdown in dental mesenchymal stem cells can inhibit cell cycle progression.

Go on to investigate the specific mechanism of PRX1^+^ dental MSCs in regulating tooth germ morphogenesis, due to the critical epithelial-mesenchymal interaction during tooth germ development, combined with the large number of previous studies of PRX1,^13^ we hypothesized that PRX1 is not only a classic marker of mesenchymal stem cells, but also contributes to the maintenance and pluripotency of dental MSCs. Then, RT-qPCR and immunofluorescence staining techniques were used to detect mRNA and protein expression levels of mesenchymal markers. Remarkably, dental MSCs lacking *Prx1* led to reduced expression of mesenchymal markers *Vim*, *N-cadherin*, and *Postn* (Fig. 4f, g). Further to validate the above results, transcriptome sequencing analysis (RNA-seq) was performed between the siPrx1 group and control group. Compared with the control group, signaling pathways regulating pluripotency of stem cells were significantly enriched in the Kyoto Encyclopedia of Genes and Genomes (KEGG) pathway network (Fig. 4h). These in vivo and in vitro experiments collectively indicated that PRX1 deficiency can induce a substantial decrease in the mesenchymal signatures, thereby inhibiting the proliferation of mesenchymal cells and affecting tooth morphogenesis, including tooth size and cusp number.

### Alternative PRX1 expression led to differential expression of *Wnt5a* morphogens

**Fig. 5:**
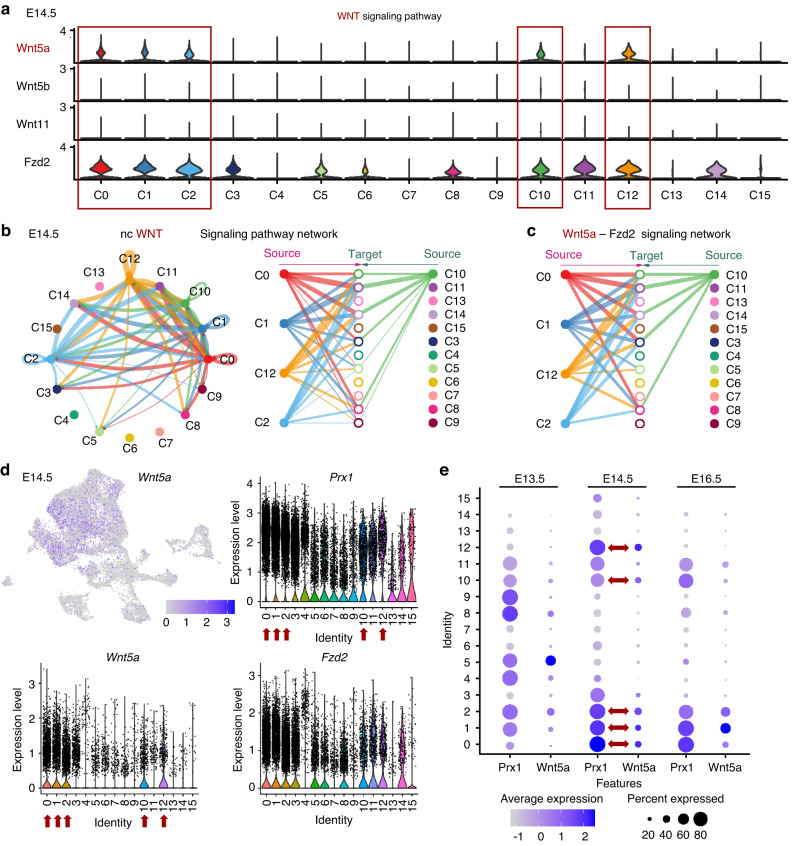
PRX1-positive stem cells functioned primarily through *Wnt5a*-mediated Wnt signaling pathway. **a** VinPlot displayed the comprehensive expression profiles of all non-canonical Wnt ligands *Wnt5a, Wnt5b, Wnt11* and highly expressed receptor *Fzd2* within varied cell clusters at cap stage (E14.5). The red boxes mark the mesenchymal subsets with high *Wnt5a* expression. **b** CellChat analysis of intercellular communication network of non-canonical Wnt signaling pathway at cap stage (E14.5). **c** CellChat analysis of intercellular communication network of Wnt5a-Fzd2 signaling pathway at E14.5. **d** UMAP and VinPlot visualization of *Wnt5a* expression and its correlation with *Prx1* at E14.5. **e** Dotplot of *Prx1* and *Wnt5a* in tooth germs at bud stage (E13.5), cap stage (E14.5) and bell stage (E16.5). *Wnt5a* had a large overlap with the PRX1^+^ mesenchymal cell subsets

For more detailed analysis of the mechanism by which PRX1 positive stem cells affect tooth germ morphology during molar development, we further analyzed the transcriptomics data. KEGG enrichment analysis also significantly enriched Wnt signaling pathway (Fig. 4h), and Differential Expression Analysis of the results of RNA-seq showed upregulated expression levels of Wnt inhibitors, such as *Wif1*, *Tcf7*, *Dkk1* and so on, in *Prx1* knockdown group. The Wnt signaling pathway works to govern a variety of events during tooth development, which make great contributions from initiation to morphogenesis.^24^ For example, mesenchymal Wnt/β-catenin signaling limits tooth number.^25^ Meanwhile, scRNA-seq data of M1 tooth germ at different developmental stages were further analyzed. Differ from most canonical Wnts (mainly *Wnt4* and *Wnt6*) enriched in Cluster 3 (C3), which represents the epithelial cell population (Fig. S4), *Wnt5a* was highly expressed in dental mesenchyme, especially in PRX1^+^ cell subclusters (Fig. 5a, Fig. S5b). CellChat analysis highlighted the important role of Wnt signaling pathway in the process of epithelial-mesenchymal interaction during tooth development. *Wnt5a*-mediated Wnt signaling in PRX1^+^ cell lineage and their daughter cells exhibited strong interaction both among themselves and with epithelial cluster (Fig. 5b, c, Fig. S5c, d), together with the KEGG enrichment analysis in *Prx1* knockdown group. Therefore, we hypothesized that PRX1-positive stem cells functioned primarily through *Wnt5a* and its downstream Wnt signaling pathway.

**Fig. 6:**
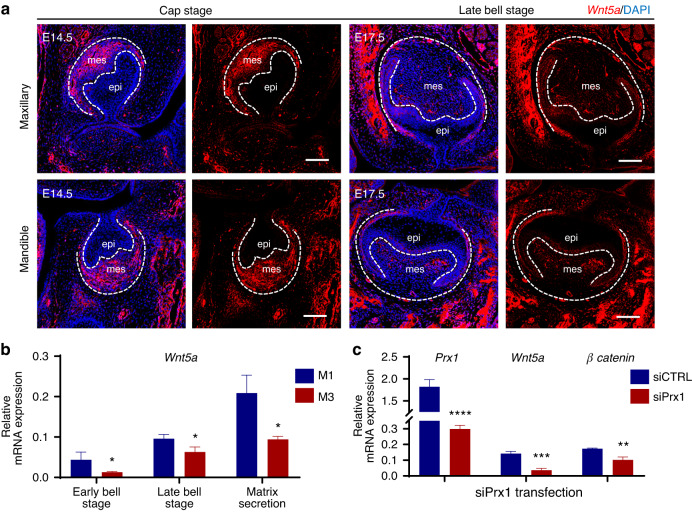
PRX1-positive stem cells induced differential *Wnt5a* expression between M1 and M3. **a** Fluorescence in situ hybridization of *Wnt5a* (red) in M1 tooth germ at E14.5 and E17.5 (*n* = 3 per developmental stages). *Wnt5a* was specifically expressed in the developing molar mesenchyme. epi epithelium, mes mesenchyme. Scale bar: 100 μm. **b** *Wnt5a* mRNA expression between M1 and M3 was evaluated by RT-qPCR (*n* = 3 per developmental stages). M3 showed significantly lower *Wnt5a* expression than control from early bell stage to matrix secretion stage. The error bar represents the standard deviation of the mean. *P < 0.05. **c** *Wnt5a* and *β-catenin* mRNA expression levels in cultured M1 cells were evaluated by RT-qPCR after *Prx1* knockdown (*n* = 3). **P < 0.01, ***P < 0.001, ****P < 0.000 1

To lend support to this hypothesis, we first preliminarily examined the correlation between PRX1-positive mesenchymal subsets and *Wnt5a* gene expression during tooth germ development stages. The results showed that *Wnt5a* was specifically expressed in the developing molar mesenchyme and had a large overlap with the PRX1^+^ mesenchymal cell populations (Fig. 5d, e, Fig. S5a). Previous studies have clarified specific expression of *Wnt5a* in dental mesenchyme rather than epithelium,^26^ which was also verified by in situ hybridization staining (Fig. 6a).Then, *Wnt5a* expression was verified between M1 and M3 using RT-qPCR. For insurance purposes we compared the expression of classical tooth germ morphogenetic molecules among first to third molars at the same tooth developmental stage as well as the same age. Other than most key morphogens (*Shh, Wnts, Bmp, Tgf-β*), *Wnt5a* showed a significantly opposite lower mRNA level in M3 tooth germ (Fig. 6b, Fig. S6). What’s more, we also examined the expression levels of *Wnt5a* and *β-catenin* in *Prx1* knockdown dental MSCs, which were significantly decreased as expected (Fig. 6c). These results further confirmed the important role of *Wnt5a*-mediated Wnt signaling in PRX1-engaged tooth morphogenesis.

### Wnt5a expression affected the morphogenesis of tooth germs

**Fig. 7:**
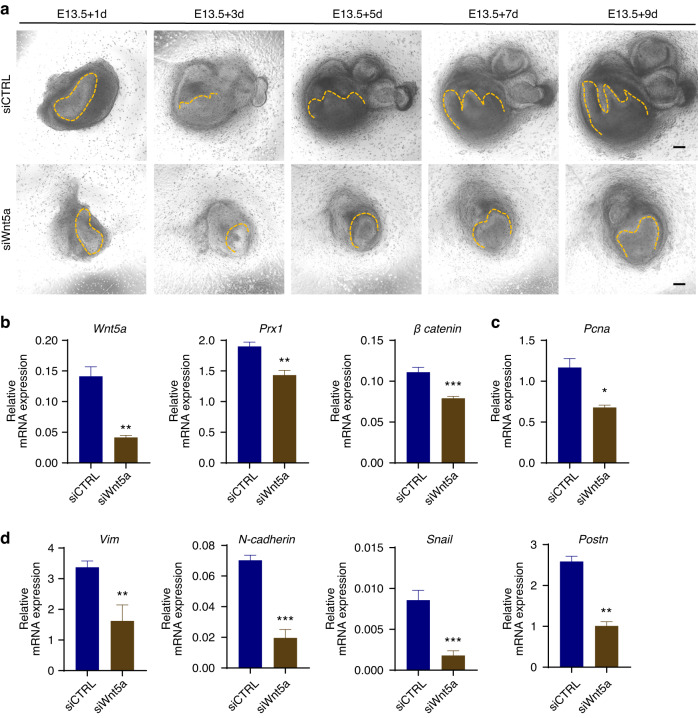
Altered *Wnt5a* expression affected M1 morphology, cell proliferation and mesenchymal pluripotency. **a** Ex vivo culture images of M1 germs at 1, 3, 5, 7 and 9 days after transfection of *Wnt5a* siRNA (siWnt5a) or negative control (siCTRL). The dotted line marks the boundary between epithelium and mesenchyme. Scale bar: 100 μm. **b** *Prx1* and *β-catenin* mRNA expression levels in cultured M1 cells were evaluated by RT-qPCR after *Wnt5a* knockdown (*n* = 3). The error bar represents the standard deviation of the mean. **P < 0.01, ***P < 0.001. **c** *Pcna* mRNA expression between siWnt5a and siCTRL group was verified by RT-qPCR (*n* = 3). it showed significantly lower *Pcna* mRNA level after *Wnt5a* knockdown. *P < 0.05. **d** mRNA expression levels of mesenchymal markers *Vim*, *N-cadherin*, *Snail* and *Postn* were evaluated by RT-qPCR after *Wnt5a* siRNA transfection (*n* = 3). **P < 0.01, ***P < 0.001

To further determine the crucial role of *Wnt5a* in PRX1^+^ dental MSCs, wildtype tooth germs were cultured in vitro and *Wnt5a* was knocked down, and the continuous observation of tooth germ morphology revealed decreased cusp number and smaller molar in the experimental group (Fig. 7a), which was consistent with morphological changes of *Prx1*-knockdown tooth germs. And the expression level of *β-catenin* in *Wnt5a* knockdown dental MSCs was significantly decreased as well (Fig. 7b). Meanwhile, the expression levels of *Pcna* and mesenchymal markers were significantly reduced compared with the negative control group (Fig. 7c, d).

**Fig. 8:**
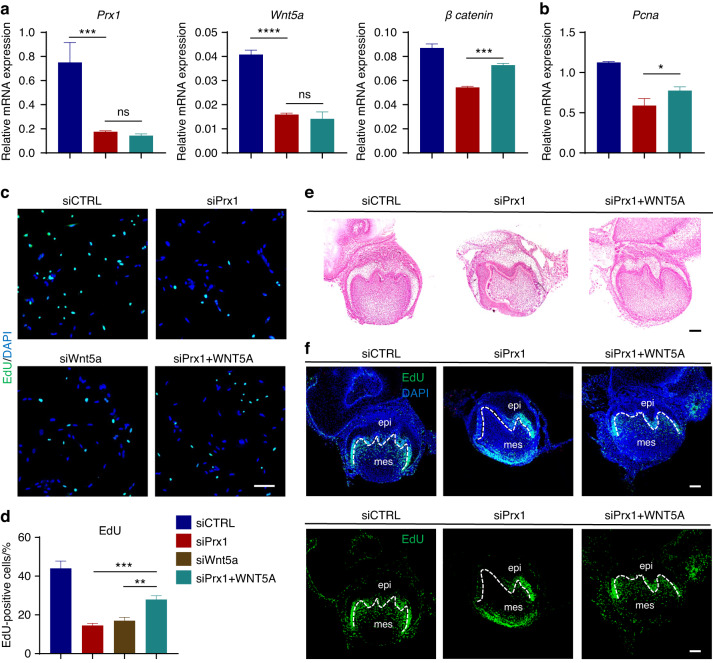
Exogenous addition of WNT5A restored M1 morphology and cell proliferation. **a** *Wnt5a* and *β-catenin* mRNA expression levels in dental mesenchymal cells were evaluated by RT-qPCR after transfection of *Prx1* siRNA (siPrx1), exogenous addition of WNT5A mimetic peptide ligand Foxy5 after *Prx1* knockdown (siPrx1 + WNT5A) and negative control (siCTRL) (*n* = 3 per group). The error bar represents the standard deviation of the mean. ***P < 0.001, ****P < 0.000 1, ns no statistical significance. **b** *Pcna* mRNA expression levels in dental mesenchymal cells were evaluated by RT-qPCR. (*n* = 3). *P < 0.05. **c** Confocal images of EdU labelled proliferating dental MSCs after transfection of *Prx1* siRNA (siPrx1), *Wnt5a* siRNA (siWnt5a), exogenous addition of WNT5A mimetic peptide ligand Foxy5 after *Prx1* knockdown (siPrx1 + WNT5A) and negative control (siCTRL) (*n* = 3 per group). Scale bar: 100 μm. **d** Quantification of EdU-positive cells in above four groups (*n* = 3). **P < 0.01, ***P < 0.001. **e** H&E staining of ex vivo cultured M1 germs at 9 days after transfection of *Prx1* siRNA (siPrx1), exogenous addition of WNT5A mimetic peptide ligand Foxy5 after *Prx1* knockdown (siPrx1 + WNT5A) and negative control (siCTRL). Scale bar: 100 μm. **f** Confocal images of EdU labelled proliferating cells in M1 germs at 9 days after transfection of *Prx1* siRNA (siPrx1), exogenous addition of WNT5A mimetic peptide ligand Foxy5 after *Prx1* knockdown (siPrx1 + WNT5A) and negative control (siCTRL) (*n* = 3 per group). The dotted line marks the boundary between epithelium and mesenchyme. epi epithelium, mes mesenchyme. Scale bar: 100 μm

As the crucial role of *Wnt5a*-mediated Wnt signaling pathway in the PRX1-positive cell population, we have also further verified whether the exogenous activation of Wnt signaling can promote proliferation and molar morphogenesis. Through the addition of a synthetic WNT5A analog ligand (Foxy5 peptide),^27^ the mRNA expression levels of *Prx1* and *Wnt5a* did not show significant changes (Fig. 8a). However, it was found that the Foxy5 peptide partially restored the suppressed expression of *β catenin* and cellular proliferation levels caused by *Prx1* knockdown (Fig. 8a–d). More importantly, the exogenous addition of Foxy5 peptide also led to the developmental reverse of the suppressed M1 morphology in PRX1 deficiency group (Fig. 8e, Fig. S7c), which is a remarkable discovery. Also, we observed the proliferation of in vitro cultured molar germs by EdU labeling, mesenchymal cells located below the future cusps of *Prx1* knockdown tooth germs exhibited significantly reduced proliferative activity, while exogenous addition of Foxy5 restored the proliferation of mesenchymal cells in this region similar to the negative control group (Fig. 8f). Taken together, these data carried out show that PRX1 positive mesenchymal cells secreted *Wnt5a* mediate tooth morphology diversity, which is expected to be a potential target for tooth morphology regulation.

## Discussion

For mammals, the variations in tooth morphology primarily manifest in the complexity of the dental occlusal surfaces, which, to a large extent, depend on the number of different tooth cusps. This complexity is functionally and physiologically significant in food chewing and digestion.^28^ Tooth cusp formation begins with the folding of the epithelial-mesenchymal junction. Previous research has identified the crucial role of enamel knots (EKs) in regulating cuspal patterning.^29,30^ The enamel knot is a cluster of cells in the dental epithelium. During tooth development, EKs act as a transient signaling center and express sets of signaling molecules, such as *Shh*, *Fgf4*, and *Bmp4*.^31^ Specially, both primary and secondary EKs are formed in molars, whereas only one EK is formed in mouse incisors. The primary EK begins to take shape at the cap stage, and later at the bell stage, the secondary EK takes form, determining the position of future cusps by directing regionally differential growth of cells around. In our research, we have observed that M1 and M3 exhibit a similar overall developmental process in the coronal aspect. However, the main distinction lies in the disparity of secondary enamel knots, resulting in varying cusps numbers. Research has been conducted to investigate the morphological differences in cusps between upper and lower molars through comparative transcriptomics, which reveals variances in the relative abundance of mesenchyme and consistent differences in gene expression within the tissues.^32^ Meanwhile, tissue engineering experiments have also proved that the shape of a heterotopic reassociation tooth depends on the origin of the mesenchyme.^10^ Therefore, it can be inferred that the mesenchyme plays a pivotal role in regulating the process of tooth morphogenesis and cusp formation.

In recent years, an expanding body of work has focused on the multifaceted subpopulations of mesenchymal stromal cells, seeking to unravel their distinct impact on tissue plasticity.^8^ The plasticity in dental mesenchymal cell types assumes paramount importance in the process driving tooth morphological determination.^19^ In case of research field on tooth regeneration, as increasing studies have focused on the heterogeneous clustering of dental mesenchymal stem/progenitor cells and their regulatory mechanisms of proliferation and differentiation,^12,33^ their unique and important transcriptional landscape and cell fate were gradually revealed, which may find new clues to regulate organ morphogenesis and regeneration. In our present study, to trace back possible reasons for the natural morphological differences between mouse M1 and M3, with the great help of scRNA-seq technology to reveal the key molecular signatures of cellular heterogeneity,^19^ we identified a significant differential expression of PRX1^+^ cell lineage between the two types of molars. Notably, the M1 tooth germ predominantly comprised mesenchymal cells of PRX1-positive lineage origin, while such cells were scarce within the M3 tooth germ. We further corroborated these findings through in vitro experiments, wherein PRX1-knockdown displayed a conspicuous alteration in first molar morphogenesis, approximating the morphology characteristic of M3.

PRX1, widely expressed in undifferentiated mesenchyme during tissue development, has garnered recognition as a marker denoting the mesenchymal stem/progenitor cell population.^34^ As substantiated by the extensive utilization of *Prx1-Cre* and *-CreER* mice across a range of MSC researches, the influential role played by PRX1^+^ mesenchymal cell subsets in dental organogenesis has been firmly established.^15,35^ Furthermore, relative study has also reported the role of *Prx1* gene in cuspal patterning, which supports our findings.^16^ Nevertheless, much remains to be elucidated concerning the precise functions and associated mechanisms attributed to PRX1 and its positive cell populations. Throughout our observation period of tooth development, we observed no notable distribution of PRX1-positive cells in M3. Both the first and third molars exhibited orderly odontogenic differentiation patterns, which suggests that more stem cells subpopulations besides PRX1 positive cells should be involved in the odontogenesis of mouse molars. However, it is important to note that the lack of PRX1-positive cells leads to significant morphological alterations and smaller developing teeth (M3) compared to the normal M1 tooth development process, which remains to be further studied.

Organogenesis and its shape in animals develop through complex coordination of cell proliferation and differentiation.^36^ Mammalian tooth germs, well-studied examples of ectodermal organ development, undergo a strict regulatory process for different morphogenesis. Thereinto, cell proliferation is regulated by various genes and signaling molecules, and finally contribute to the future tooth formation.^37,38^ Abnormal expression of crucial cell cycle regulators, such as cyclin D1 and P21, can disrupt the normal progression of the cell cycle, thereby affecting the proliferative activity of epithelial or mesenchymal cells and ultimately altering the pattern and size of teeth.^39,40^ In our research, we observed a significant difference in the number of proliferating cells between M1 and M3, and the distribution of these cells is closely associated with PRX1-positive cells and their progeny. So, based on the above, we postulated that differences in proliferative capacity might underlie the morphological disparity between PRX1-positive and PRX1-negative dental MSCs. Accordingly, we conducted a series of investigations to validate our hypothesis. In vivo, there were disparities in proliferation between M1 and M3 mesenchymal tissues. PRX1-positive dental MSCs are in a more actively proliferative state, which promotes M1 molar morphogenesis. In vitro studies revealed slower proliferation and cell cycle arrest following *Prx1* knockdown. Therefore, modulating the proportion of PRX1-positive dental MSCs may offer potential avenues for regulating tooth shapes.

**Fig. 9:**
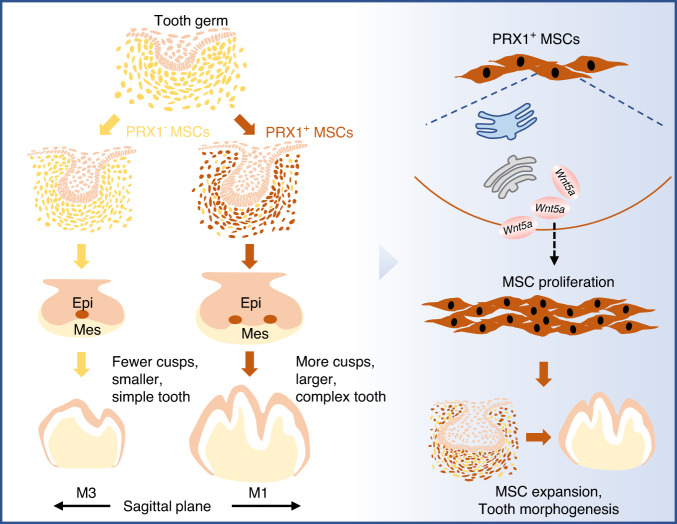
Schematic diagram of PRX1-positive MSCs functions in regulating molar morphogenesis. PRX1-positive dental MSCs regulates mesenchymal plasticity and cell proliferation by promoting *Wnt5a* expression, thereby drives diverse molar shape. epi epithelium, mes mesenchyme

The crucial question then becomes: how do PRX1-positive cells impact proliferation? During mammalian development, the process of generating repetitive structures such as digits, vertebrae, and teeth relies on the involvement of morphogens.^4,41^ Notably, morphogens form distinct morphogenetic gradients in growing tissues, which play a crucial role in limiting tissue patterning.^42^ It is likely that some of these morphogens contribute to the variations observed in tooth morphogenesis. WNT5A, a member of the evolutionarily conserved noncanonical Wnt ligand protein family, has been established as essential in various developing organs, including the lung, blood vessels, cartilage, and tooth.^43–45^ *Wnt5a* exhibits robust expression in the dental mesenchyme during the early stages of tooth development.^26,33^ Based on our single-cell data showing a high correlation between PRX1-positive cells and *Wnt5a* expression, we attribute the morphological disparities seen in PRX1-positive and PRX1-negative dental cell populations to differential expression patterns of the *Wnt5a* morphogen. Knockdown of *Wnt5a* showed a reduction in cusp number and smaller molar, consistent with the phenotype observed in PRX1 deficiency group. While adding the WNT5A mimetic peptide ligand Foxy5 markedly up-regulated MSC proliferation and reversed the M1 morphology suppressed due to PRX1 deficiency. Experimental evidence from targeted mutation studies in mice also supports the idea that disruption of *Wnt5a* hampers the balance between cell proliferation and death, resulting in abnormally patterned cusps and smaller teeth.^46,47^ Recent investigations in mouse incisors propose that *Wnt5a*-mediated noncanonical Wnt signaling contributes to the regulation of GLI1^+^ MSCs, thereby influencing their maintenance.^48^ We also looked at the canonical Wnt signaling pathway, the CellChat analysis for intercellular communication network revealed that canonical Wnt signaling may mainly originate from epithelial cell populations and interact with epithelial and mesenchymal cell populations during morphogenesis, which is consistent with previous study,^49,50^ suggesting that canonical Wnt signaling is crucial for maintaining the dental epithelial stem cell niche. These findings, combined with our own results, bolster the notion that the *Wnt5a*-mediated Wnt signaling pathway governs molar morphogenesis by modulating the proliferation of PRX1^+^ dental MSCs (Fig. 9).

In summary, PRX1-positive cells exhibit active proliferation, ultimately leading to the striking different molar morphogenesis between M1 and M3. Furthermore, *Wnt5a*, as a highly expressed morphogen gene in PRX1-positive cells, plays a critical role in this process. Collectively, our findings provide evidence that PRX1-positive dental MSCs regulates mesenchymal plasticity and cell proliferation by promoting Wnt5a expression, thereby drives diverse molar shape. Consequently, this regulatory mechanism underlies the process of molar morphogenesis in mice.

## Materials and methods

### Animals

All animal experiments were approved by the Animal Welfare Committee of the Affiliated Stomatology Hospital of Tongji University ([2022]-DW-06). Mouse experiments were performed in accordance with the National Institutes of Health’s Guide for Care and Use of Laboratory Animals. This study conformed to the ARRIVE Guidelines. Transgenic *Prx1-Cre*, *Prx1CreER-GFP* and *Osx-Cre* mice and *R26R*^*tdTomato*^ mice were used in this study. Those transgenic mice in a background of C57BL6/J were generated by CRISP/Cas9-based approach. Both wildtype and transgenic mice were maintained in a specific pathogen-free (SPF) facility under a 12:12-h day/night illumination cycle. Animals were euthanized by cervical dislocation after inhalation anesthesia.

### Lineage tracing

The lineage tracing lines were crossed to *R26R*^*tdTomato*^ respectively, and the cytoplasmic red marks the Cre event and progeny of corresponding positive cells in the mice.

### Ex vivo culture and transfection of embryonic tooth germs

Tooth germs were rapidly dissected from E13.5 mouse embryos with the help of precision tweezers and research inverted system microscope, and placed on cell culture inserts (6 wells, Falcon). The mandible molars were cultured at 37 °C in a humidified atmosphere of 5% CO_2_ in specific organ culture medium as previously published.^22,23^ After that, siRNA transfection mixture was prepared using PEI transfection reagent (Proteintech, Wuhan, China), and then dropped on the top of every tooth germ. In the experiment group, Foxy5 peptide (MedChemExpress, USA) or scrambled peptide solution was added directly to the culture medium after siRNA transfection. For observations, photos were taken under microscope every 2 days. Both siRNA and organ culture medium must be refreshed every 2 or 3 days.

The synthetic sequences of siRNAs are as follows:

siPrx1: 5′-GGGACAGCCUCUCCGUACATT-3′;

siWnt5a: 5′-CCUGUUCAGAUGUCAGAAGUATT-3′;

Negative control (siCTRL): 5′-UUCUCCGAACGUGUCACGUTT-3′.

### Isolation and culture of mouse dental MSCs

Tooth germs were dissected from E13.5 mouse embryos and digested by type I collagenase. The epithelium was separated from the mesenchymal tissues. The dental MSCs were cultured in a mixed culture medium containing α-minimum essential medium (α-MEM, HyClone, USA), 10% fetal bovine serum (FBS, Gibco, USA), 100 U/mL penicillin, and 100 μg/mL streptomycin (Gibco, USA) at 37 °C with a humidified atmosphere of 5% CO_2_. The culture medium must be changed every 2 or 3 days. When reaching a confluency of 90%, the cells were suitable for passage. P3-P5 cells were used in this experiment.

### Tissue processing and histological staining

Mandible tissues and molars of different development stages were processed and fixed in 4% paraformaldehyde (PFA) for 48 h. Postnatal samples were decalcified in 0.1% DEPC-treated 10% ethylene diamine tetraacetic acid (EDTA) (pH 7.4) at 4 °C for different days, which varied with sample size and age. Then, after serial processes of dehydration, transparency and wax immersion, tissues were embedded in paraffin. All these mandible tissues were sectioned at 4 μm for Hematoxylin & Eosin (H&E) staining with standard protocols.

### Immunofluorescence staining

For immunofluorescence, decalcified samples were embedded in optimal cutting temperature compound (OCT) after dehydration, and cut at 8 μm sections. Slices were treated with 3% hydrogen peroxide for 15 min and blocked with heat inactivated goat serum before incubation in primary antibodies overnight at 4 °C. The following primary antibodies were used for detection: anti-PRX1 (1:100, Santa Cruz Biotechnology, Dallas, TX), anti-Osterix (1:200; Abcam, Cambridge, UK), anti-RUNX2 (1:200; Abcam, Cambridge, UK), and anti- Vimentin (1:100, Cell Signaling Technology, Boston, USA). After incubation with Alexa Fluor 488 IgG or 594 IgG (1:100 0, Invitrogen, USA) at room temperature for 1 hours, the cell nuclei were counterstained with DAPI (1 μg/mL, Sigma-Aldrich, USA). Immunofluorescence images were visualized and captured with a confocal laser scanning microscope (Nikon, TI2-E + A1 R, Japan).

### Edu cell proliferation assay

Wildtype mice at different M1 or M3 development stages were i.p. injected with EdU (5-ethynyl-2’-deoxyuridine, Beyotime, Shanghai, China) 2 h prior to killing and tissues were isolated and processed as described. Different treated mouse dental MSCs were added with EdU 12 h before sample collection. And this assay was performed with a BeyoClick™ EdU-488 Kit (Beyotime, Shanghai, China) according to the manufacturer’s protocol.

### Cell apoptosis assay

Mandible tissues and molars of wildtype mice at corresponding development stages of M1 and M3 were collected and embedded in paraffin. The apoptotic cell distribution was examined using a TUNEL Apoptosis Detection Kit (Yeasen Biotech, China) according to the manufacturer’s instructions.

### RNA sequencing and quantitative real-time reverse transcription polymerase chain reaction (RT-qPCR)

For RNA sequencing (RNA-seq), the lower first molar germs were dissected from E13.5 mouse embryos and cultured in vitro. After siRNA transfection, tooth germs were collected and subjected to RNA extraction (TaKaRa, Beijing, China) for complementary DNA (cDNA) library preparation and sequencing.

For RT-qPCR, total RNA from mouse molars or dental MSCs was extracted with Trizol Reagent (Invitrogen, USA) according to the manufacturer’s instructions. cDNA was synthesized from 1 µg total RNA using a reverse transcript kit (TaKaRa, Beijing, China). RT-qPCR was performed with SYBR Premix Ex Taq II (TaKaRa, Beijing, China). The primer sequences were listed in the Appendix Table S1. Relative gene expression was normalized to the expression of the house-keeping gene *Gapdh*.

### Flow cytometry

Mouse dental MSCs, both treated with siPrx1 transfection and the negative control group (siCTRL), were collected from the culture plates and then fixed with 70% alcohol. Propidium staining was followed by the instructions of Cell Cycle and Apoptosis Analysis Kit (Beyotime, Shanghai, China). Flow cytometry analysis was performed using a BD LSR Fortessa (BD, USA) and BD FACSDiva software.

### In situ hybridization

Mandible tissues and molars of different development stages were processed and fixed in 4% paraformaldehyde (PFA) with water-diethylpyrocarbonate (DEPC) treated. Immunostaining was performed with 8 µm paraffin samples and hybridized with digoxygenin-labelled RNA probes. And this assay was performed with a *Wnt5a* mRNA ISH Kit (Boster, China) according to the manufacturer’s protocol.

In situ Hybridization of *Prx1* was also performed using an RNAscope™ 2.5 LS ReagentKit RED kit (Advanced Cell Diagnostics, USA) according to the manufacturer’s protocol.

### Re-analysis of scRNA-seq data

The scRNA-seq data were obtained from the GEO database (GSE189381).^12^ The analysis was performed using Seurat v4.0.5 and R version 4.0.4. Clusters were visualized using Uniform manifold approximation and projection (UMAP). Published markers for the dental mesenchyme and epithelium identified the dental mesenchymal and epithelial cell populations in the mouse molar. CellChat was implemented by Seurat to evaluate the potential for cell-cell interactions in E13.4 and E14.5 datasets. Any subsequent analysis was done using raw data.

### Statistical analysis

All data were analyzed by GraphPad Prism 8.0 software and expressed as mean ± standard deviation (SD). Statistical differences were evaluated using Student’s *t*-test or one-way analysis of variance (ANOVA). *P* < 0.05 was considered as significant statistically differences.

### Supplementary information

Revised supplemental information

## Data Availability

The raw and processed scRNA-seq data used in this study have been deposited in the Gene Expression Omnibus (GEO) database with accession number: GSE189381. All other data in this study are available from the corresponding author upon reasonable request.
