# Supplementary material for: PRX1-positive mesenchymal stem cells drive molar morphogenesis
Source: Int J Oral Sci. 2024 Feb 19;16:15. doi: 10.1038/s41368-024-00277-0 (PMC10874978; doi:10.1038/s41368-024-00277-0)
Supplement: Supplementary file 1 — Revised supplemental information [file 41368_2024_277_MOESM1_ESM.docx]

**Supplemental information**

**Title: PRX1-positive Mesenchymal Stem Cells Drive Molar Morphogenesis via *Wnt5a*-mediated Cell Proliferation**

**Authors:** Xiaoqiao Xu, Xuyan Gong, Lei Zhang, Han Zhang, and Yao Sun

**Figure S1**

**Figure S1. Dental PRX1 Expression Through Tooth Development Stages.** (a) Immunofluorescence for PRX1 (green) in the tooth germs of first molar at different developmental stages of the embryo. PRX1 protein highly expressed in the mesenchyme of M1 tooth germs. The dotted line marks the boundary of epithelium and mesenchyme, respectively. epi epithelium, mes mesenchyme. Scale bar: 100 μm. (b) Immunofluorescence for PRX1 (green) in M1 and M3 at postnatal 0.5 days (P0.5) and postnatal 7.5 days (P7.5). PRX1 protein rarely expressed in either M1 or M3 tooth after birth. od odontoblast, am ameloblast, dp dental pulp. Scale bar: 100 μm.

**Figure S2**

**Figure S2. Comparison of corresponding development periods between M3 and M1.** (a) H&E staining of tooth germs in the coronal plane showing the morphological differences between M1 and M3 during development. Scale bar: 100 μm. (b) *Prx1* mRNA expression levels throughout the first molar development were evaluated by RT-qPCR. The error bar represents the standard deviation of the mean. *****P* < 0.0001. (c) *Prx1* mRNA expression differences between M1 and M3 was verified by RT-qPCR. M3 showed significantly lower *Prx1* expression both in early and late bell stage. **P* < 0.05, ****P* < 0.001.

**Figure S3**

**Figure S3. PRX1 deficiency in Third Molar did not affect differentiation of dental MSCs.** (a) Co-staining of OSX^+^ lineage cells (red) and odontogenic differentiation marker OSX (green) in both M1 and M3 of 2-week-old mice. od odontoblast, am ameloblast, dp dental pulp. Scale bar: 100 μm. (b) Immunofluorescence for odontogenic differentiation marker RUNX2 (green) in M1 and M3 of 2-week-old mice. Scale bar: 100 μm.

**Figure S4**

**Figure S4. CellChat analysis identified the intercellular communication network of canonical Wnt signaling pathway.** (a) VinPlot displayed the comprehensive expression profiles of all canonical Wnt ligands (especially *Wnt4* and *Wnt6*) within varied cell clusters at bud stage (E13.5). (b) CellChat analysis of intercellular communication network of canonical Wnt signaling pathway at bud stage (E13.5).

**Figure S5**

**Figure S5. ScRNA-seq analysis identified the intercellular communication network of *Wnt5a*.** (a) UMAP and VinPlot visualization of *Wnt5a* expression and its correlation with *Prx1* at E13.5 and E16.5. (b) VinPlot displayed the comprehensive expression profiles of all non-canonical Wnt ligands *Wnt5a, Wnt5b, Wnt11* and highly expressed receptor *Fzd2* within varied cell clusters at bud stage (E13.5). The red boxes mark the mesenchymal subsets with high Wnt5a expression. (c) CellChat analysis of intercellular communication network of non-canonical Wnt signaling pathway at bud stage (E13.5). (d) CellChat analysis of intercellular communication network of Wnt5a-Fzd2 signaling pathway at E13.5.

**Figure S6**

**Figure S6. Classical Morphogens expression between molars.** mRNA expression levels of classical Morphogens, including *Wnt3a*, *Wnt7a*, *Wnt10a, Shh*, *Bmp2*, *Bmp4*, *β-catenin*, and *Tgf β*, as well as *Yap1*, were evaluated by RT-qPCR between M1 and M3 at similar developmental stages (*n* = 3). Morphogens expressed in M3 of P12.5 mice were compared to Morphogens expressed in M1 of P5.5 mice. The error bar represents the standard deviation of the mean. ns, no statistical significance, **P* < 0.05, ****P* < 0.001.

**Figure S7**

**Figure S7. Transfection efficiency and morphological changes of tooth germs cultured in vitro.** (a) Representative images of ex-culture tooth germs with FITC tagged scrambled siRNA added showing the transfection levels of siRNAs. Scale bar: 100 μm. (b) *Prx1* mRNA expression levels in the ex-culture tooth germs between siPrx1 and siCTRL group was verified by RT-qPCR (*n* = 3). it showed significantly lower *Prx1* expression after *Prx1* knockdown. ****P* < 0.001. (c) Ex vivo culture images of M1 germs at 1, 3, 5, 7 and 9 days after transfection of *Prx1* siRNA (siPrx1), exogenous addition of WNT5A mimetic peptide ligand Foxy5 after *Prx1* knockdown (siPrx1+WNT5A) and negative control (siCTRL) (*n* = 3 per group). The dotted line marks the boundary between epithelium and mesenchyme. Scale bar: 100 μm.

**Appendix Table S1.** Primer sequences for RT-qPCR.

| Gene name | Forward primer (5’-3’) | Reverse primer (5’-3’) |
| --- | --- | --- |
| *Gapdh* | TGTGTCCGTCGTGGATCTGA | CCTGCTTCACCACCTTCTTGA |
| *Prx1* | ACTTCTCCGTCAGTCACCTGCTAG | CTCGCCCACACTTTCGTCTGC |
| *Pcna* | AGATGTGCCCCTTGTTGTAGAG | GAAAAGACCTCAGGACACGCT |
| *Vim* | AAGCACCCTGCAGTCATTCAGA | GCAAGGATTCCACTTTCCGTTC |
| *N-Cadherin* | ATGCTGACCACTCTCACTGCTC | ACATTTGGCGACTCTCTGTCC |
| *Postn* | GGTGTCCTAGAAAGGATCATGG | CAGAGCACTGGAGGGTATTTAG |
| *Snail* | GAGGACAGTGGCAAAAGCTC | TCGGATGTGCATCTTCAGAG |
| *Wnt5a* | GGACGCTAGAGAAAGGGAACGAATC | GCCAGACACTCCATGACACTTACAG |
| *β-catenin* | AGGAATGAAGGCGTGGCAACATAC | GGCACCAATGTCCAGTCCAAGATC |
| *Wnt3a* | CAAGTATGACAGTGCCTCGGAGATG | GGGTTAGGTTCGCAGAAGTTGGG |
| *Wnt7a* | CTGGGTGCGAGCATCATCTGTAAC | GCCATTTCGGAACTGAAACTGACAC |
| *Wnt10a* | CCATGAGTGCCAGCATCAGTTCC | AACCGCAAGCCTTCAGTTTACCC |
| *Shh* | AAAGCTGACCCCTTTAGCCTA | TTCGGAGTTTCTTGTGATCTTCC |
| *Bmp2* | GCTTCTTAGACGGACTGCGG | GCAACACTAGAAGACAGCGGGT |
| *Bmp4* | CTTCAACCTCAGCAGCATCC | GATGAGGTGTCCAGGAACCA |
| *Tgf β* | GCTGAACCAAGGAGACGGAATAC | GCCATGAGGAGCAGGAAGGG |
| *Yap1* | GAGAAGGAGAGACTGCGGTTGAAAC | CCCTCCATCCTGCTCCAGTGTAG |
